# Associations between genetic variants of Toll-interacting proteins and interstitial lung diseases: a systematic review and meta-analysis

**DOI:** 10.1186/s13023-024-03410-8

**Published:** 2024-11-22

**Authors:** Xiaoyuan Li, Beibei Cui, Lili Jiang

**Affiliations:** 1 Department of Pathology, West China Hospital, Sichuan University, Chengdu, Sichuan China; 2 Department of Rheumatology and Immunology, West China Hospital, Sichuan University, Chengdu, China

**Keywords:** *TOLLIP*, Genetic variants, Interstitial lung disease, Idiopathic pulmonary fibrosis, Meta-analyses

## Abstract

**Background:**

Genetic polymorphisms in Toll-interacting protein (*TOLLIP*) have been documented in relation to clinical manifestations of interstitial lung disease (ILD). Nevertheless, the findings across studies present inconsistencies. The present meta-analysis endeavors to elucidate the nexus between genetic variations in *TOLLIP* and the onset and prognosis of interstitial lung disease (ILD), with the overarching aim of providing insight into the pathophysiological underpinnings of ILD.

**Method:**

This systematic review was registered in PROSPERO. The OVID MEDLINE, OVID EMBASE, and Web of Science electronic databases were searched.

**Results:**

Fourteen studies with a total of 4821 cases and 9765 controls were examined. The final *TOLLIP* variants to be included in this meta-analysis were rs5743890, rs111521887, and rs3750920. There were significantly fewer *TOLLIP* rs5743890 minor allele C carriers among individuals with interstitial lung disease (ILD) than among those without this condition (11.42% vs. 18.92%). Conversely, patients with ILD exhibited higher frequencies of rs111521887 minor allele G carriers (28.92% vs. 22.44%) and rs3750920 minor allele T carriers (40.06% vs. 34.00%). A potential association between rs5743890_C and a reduced incidence of ILD was plausible (*p* = 0.04, OR = 0.72, 95% CI = 0.53–0.99). Furthermore, a stratified analysis revealed that rs5743890_C was significantly associated with a decreased risk of IPF (*p* = 0.004, OR = 0.62, 95% CI = 0.44–0.86). There was a significant correlation between susceptibility to ILD and rs111521887 G (*p* < 0.00001, OR = 1.48, 95% CI = 1.33–1.65) and rs3750920 T (*p* < 0.00001, OR = 1.34, 95% CI = 1.26–1.44). The survival of IPF patients was correlated with the *TOLLIP* rs5743890 SNP, and patients with the rs5743890_C genotype had worse survival (*p* = 0.02, HR = 1.59, 95% CI = 1.07–2.36).

**Conclusion:**

This study showed that rs5743890_C was associated with a lower incidence of ILD and a worse survival rate in patients with IPF. Rs111521887_G and rs3750920_T were found to be associated with an elevated risk of ILD incidence, while no significant association was observed with ILD prognosis. Furthermore, studies are warranted to validate our results and assess the effects of *TOLLIP* genetic variants on ILD.

**Supplementary Information:**

The online version contains supplementary material available at 10.1186/s13023-024-03410-8.

## Introduction

Interstitial lung diseases (ILDs) are a heterogeneous group of pulmonary diseases characterized by fibrosis and inflammation in the pulmonary parenchyma [1]. Fibrotic ILDs, such as idiopathic pulmonary fibrosis (IPF), chronic hypersensitivity pneumonitis (CHP), and connective tissue disease-associated ILD (CTD-ILD), are usually associated with severe morbidity and early mortality.

Emerging evidence has revealed a genetic basis for the crucial role of the ILD incidence. The functions of identified susceptibility genes in ILD vary from host defence to cell‒cell adhesion to DNA repair [2]. These mutations account for only a small fraction of the risk of developing idiopathic interstitial pneumonia, and there has been no genetic variation associated with ILD results. In later studies, the Single nucleotide polymorphisms (SNPs) of some genes are reportedly associated with clinical features of lung fibrosis. The *MUC5B* rs35705950 SNP is a common and strong genetic risk factor for ILD in the general population and has been previously meta-analysed [3].

Toll-interacting protein (*TOLLIP*), which can inhibit the signaling of the TOLL-like receptor and the production of tumor necrosis factor-a (TNF-a) and IL-6, is involved in various diseases, such as Parkinson’s disease, Alzheimer’s disease, inflammatory bowel disease, myocardial hypertrophy, and idiopathic pulmonary fibrosis [4–9]. Note that *TOLLIP* can regulate the trajectory of lung disease in both positive and negative ways. Genetic variants of *TOLLIP* have been demonstrated to be associated with various chronic lung diseases, including idiopathic pulmonary fibrosis, asthma, primary graft dysfunction following lung transplantation, and pulmonary infections [9–12]. In 2013, a genome-wide association study led by Imre Noth et al., published in Lancet Respiratory Medicine, first identified variants in the *TOLLIP* gene as being associated with susceptibility to IPF and increased mortality, highlighting the gene’s significance in the disease’s pathogenesis [18]. Subsequent research in 2015 by Justin M. Oldham et al. revealed a significant interaction between *TOLLIP* SNPs and N-acetylcysteine (NAC) treatment, suggesting that further investigation into *TOLLIP* SNPs could offer new avenues for therapeutic strategies in IPF [8]. Located in proximity on chromosome 11p15.5, *TOLLIP* and MUC5B are crucial in the immune response, bronchial, and alveolar fibrosis. While both genes are involved in host defines, *TOLLIP* is particularly notable for its association with inflammatory signalling. However, studies on *TOLLIP* genetic variants and ILD have yielded conflicting results. To address this, we conducted a meta-analysis to examine the relationship between *TOLLIP* genetic variants and both the incidence and prognosis of ILD. Our aim was to clarify the role of these variants in the pathogenesis of ILD and to identify potential new avenues for therapeutic strategies.

## Method

### Study registration

The systematic review was registered in PROSPERO (CRD42022308659). We followed the Preferred Reporting Items for Systematic Reviews and Meta-Analyses 2009 statement.

### Search strategy

To perform systematic retrieval, we searched the electronic databases OVID MEDLINE, OVID EMBASE, and Web of Science using the Medical Subject Headings term and a keyword on Jan 30, 2022. The search terms used were as follows: “interstitial lung diseases”, “ILD”, “genetic variation”, “variant”, “gene polymorphism”, “*TOLLIP*”, and “Toll-interacting protein”. The detailed search strategy is provided in the supplemental file. The duplication was removed. The references of retrieved publications were manually filtered for potentially relevant articles.

### Inclusion criteria and exclusion criteria

The inclusion criteria were as follows: (a) contained original data and (b) provided adequate data to calculate odds ratios (ORs) and 95% confidence intervals (CIs). The exclusion criteria were as follows: (a) had overlapping data or (b) had family members studied because the analyses were based on linkage considerations. (c) English texts were not available. (d) Abstracts, reviews, comments and conference presentations.

### Data extraction and quality assessment

The titles and abstracts of the retrieved studies were filtered based on our study selection criteria. The full texts of the remaining studies from the first screening were downloaded for further screening based on the study eligibility criteria. Two authors independently screened the studies, and any disagreements were resolved via discussion or adjudication by a third reviewer, if necessary.

Two authors independently collected data on the first author’s family name, year of publication, country, study design, ethnicity, number of participants, classification of ILD, allele frequency, hazard ratio (HR) and 95% confidence interval (CI) of survival associated with minor alleles. If a study contained several independent groups, the groups were listed.

The methodological quality of the included studies was assessed using the Newcastle–Ottawa quality assessment scale (NOS). A total of three domains—selection, comparability, and exposure—with eight numbered items yielded the highest total score of 9. For selection and exposure, each of seven numbered items was scored as 1 if the answer was yes, while for comparability, a maximum score of 2 was given for a numbered item. Studies with a score ≥ 6 were considered high-quality studies. Two authors performed the methodological quality assessment, and any disagreements were resolved via discussion or adjudication by a third reviewer, if necessary.

### Statistical analysis

We performed the data analyses using RevMan software (version 5.4). Meta-analyses were performed to explore the associations between *TOLLIP* genotypes and susceptibility to and prognosis of ILD patients. Subgroup analysis was performed according to the classification of ILD. Time-to-event data were incorporated into the meta-analysis by the methods published by Jayne F Tierney et al. [13].

The effect size of the meta-analysis was estimated by incidence with 95% CIs and odds ratios (ORs). We assessed clinical diversity across studies through statistical heterogeneity using I^2^ and p values. I^2^ values of 25%, 50%, and 75% represented low, moderate, and high heterogeneity, respectively. Fixed-effect models (FEMs) were used for synthetic analyses. A random effects model was applied when heterogeneity was greater than 50% or *P* < 0.05. Sensitivity analysis was performed by excluding studies one by one to identify the potential source of heterogeneity. We assessed the risk of publication bias via funnel plots.

## Results

### Literature search and study characteristics

**Fig. 1:**
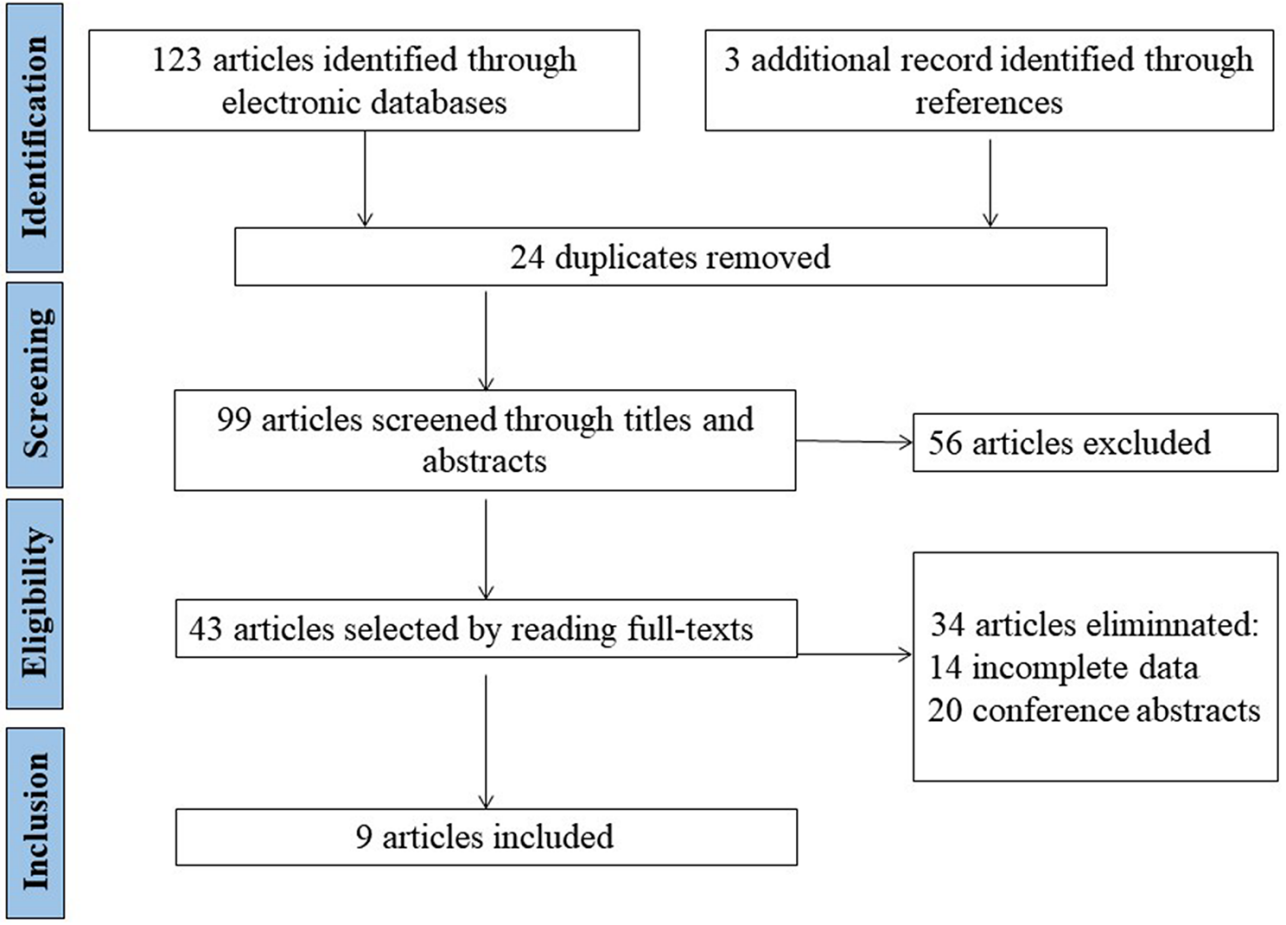
Study selection flowchart

A total of 99 articles were retrieved via electronic and manual searching after removing duplications, with 43 selected articles for further evaluation based on title and abstract details. Of all the selected articles, 20 were meeting abstracts, and 14 lacked essential data; thus, these 34 articles were excluded. Among the 9 included articles, 3 studies contained complete data from two or three independent cohorts; thus, we listed them. A total of 14 eligible studies were ultimately analysed, involving 4821 patients and 9765 controls. The details are shown in Fig. 1. The characteristics of the eligible studies are shown in Tables 1 and 2.

**Table 1:** Characteristics of individual studies on *TOLLIP* variants included in the meta-analysis of susceptibility to ILD

References	Year	Country	Ethnicity	Disease	Numbers		Minor alleles (%)		Allele association			
					Case	Control	Case	Control	OR	95% CI		*p* value
**rs5743890**												
Patrícia Caetano Mota^25^	2022	Portuguese	European	IPF	64	74	14.80	18.20	0.78	0.41	1.48	0.45
Javier Guzmán-Vargas^14^	2021	Mexico	European	IPF	93	174	0.00	41.52	0.01	0.00	0.10	0.00
Jonsson, E. ^15^	2021	Sweden	European	RA-ILD	60	2350	20.00	19.42	1.04	0.63	1.70	0.01
Francesco Bonella^16^	2021	Germany	European	IPF	62	50	11.29	11.00	1.03	0.45	2.38	0.95
Brett Ley^17^	2017	America	European	CHP/IPF	442	503	15.27	14.20	1.09	0.84	1.4	0.51
Imre Noth-1^18^	2013	America	European	IPF	542	542	10.98	15.04	0.70	0.54	0.90	0.01
Imre Noth-2^18^	2013	America	European	IPF	544	687	9.00	14.99	0.56	0.43	0.72	0.00
Imre Noth-3 ^18^	2013	America	European	IPF	324	702	10.03	17.02	0.54	0.41	0.73	0.00
In all					2131	5082	11.42	18.92				
**rs111521887**												
Patrícia Caetano Mota^25^	2022	Portuguese	European	IPF	64	74	19.50	12.80	1.65	0.86	3.16	0.13
Javier Guzmán-Vargas^14^	2021	Mexico	European	IPF	93	174	43.75	44.23	0.98	0.47	2.04	0.96
Jonsson, E.^15^	2021	Sweden	European	RA-ILD	60	2350	33.33	19.55	2.06	1.4	3.03	0.00
Imre Noth-1^18^	2013	America	European	IPF	542	542	28.97	21.03	1.53	1.26	1.86	0.00
Imre Noth-2^18^	2013	America	European	IPF	544	687	25.00	17.98	1.52	1.25	1.85	0.00
Imre Noth-3^18^	2013	America	European	IPF	324	702	22.99	19.02	1.27	1.01	1.59	0.04
In all					1627	4529	28.92	22.44				
**rs3750920**												
Patrícia Caetano Mota^25^	2022	Portuguese	European	IPF	64	74	50.00	41.20	1.43	0.89	2.3	0.14
Francesco Bonella^16^	2021	Germany	European	IPF	62	50	45.97	46.00	1.00	0.59	1.69	1.00
Fingerlin, T. E.^19^	2013	America	European	IIP	1615	4683	50.19	43.80	1.29	1.19	1.40	0.00
Imre Noth-1^18^	2013	America	European	IPF	542	542	29.15	21.03	1.54	1.27	1.88	0.00
Imre Noth-2^18^	2013	America	European	IPF	544	687	25.00	17.98	1.52	1.25	1.85	0.00
In all					2827	6036	40.06	34.00				

OR: odds ratio; CI: confidence interval; IPF: idiopathic pulmonary fibrosis; RA: rheumatoid arthritis; ILD: interstitial lung diseases; IIP: idiopathic interstitial pneumonia; CTD-ILD: connective tissue disease associated interstitial lung diseases; IPAF: Interstitial pneumonitis with autoimmune features; CHP: chronic hypersensitivity pneumonitis

**Table 2:** Analysis of the association between the TOLLIP variants and susceptibility to ILD

Variants	Population	No. of studies	No. of alleles		Test of association			Test of heterogeneity		
			Case	Control	OR	95%CI	*p* value	Model	*p* value	I^2^
rs5743890, C	Overall	8	4170	10,147	0.72	0.53-0.99	0.04	RE	<0.0000	80%
	IPF	6	3186	4440	0.62	0.44-0.86	0.004	RE	0.004	71%
rs111521887, G	Overall	6	3100	9022	1.48	1.33-1.65	<0.0000	FE	0.3	17%
rs3750920, T	Overall	5	5654	12,072	1.34	1.26-1.44	<0.0000	FE	0.23	29%

OR: odds ratio; CI: confidence interval; RE: R random effects model; FE: F fixed effects model

### Methodological quality

Of the 9 articles, 8 had an NOS score ≥ 6, indicating high study quality, while one was rated as being of low quality (NOS score < 6). The NOS scores are shown in supplemental Table S1.

All the selected studies were thoroughly analysed. The three *TOLLIP* variants rs5743890, rs111521887 and rs3750920 were ultimately included in this meta-analysis.

### Allele frequency of the *TOLLIP* variants

The frequency of Rs5743890_C was lower in patients with ILD than in controls (11.42% vs. 18.92%). In contrast, the frequencies of rs111521887_G (28.92% vs. 22.44%) and rs3750920_T (40.06% vs. 34.00%) were greater in patients with ILD. The details are shown in Table 1.

### Meta-analysis of the *TOLLIP* minor alleles and susceptibility to ILD

**Fig. 2:**
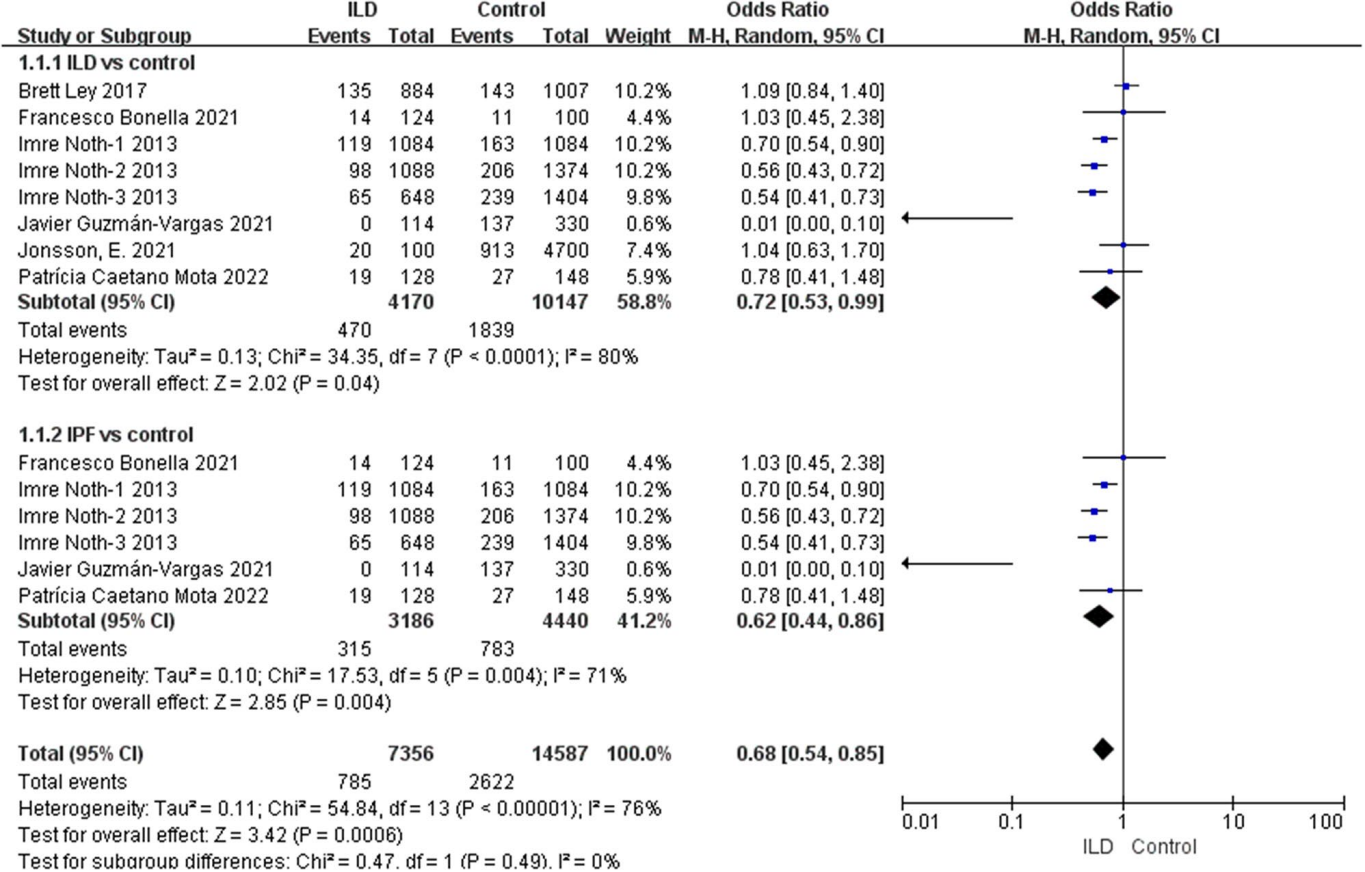
Analysis of the association between rs5743890_C and susceptibility to ILD

**Fig. 3:**
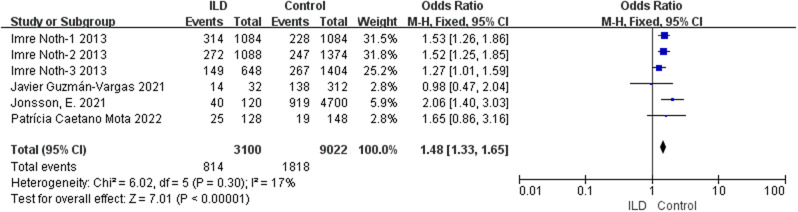
Analysis of the association between rs111521887_G and susceptibility to ILD

**Fig. 4:**
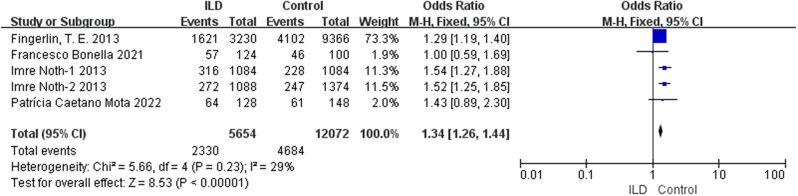
Analysis of the association between rs3750920_T and susceptibility to ILD

According to our comprehensive meta-analysis encompassing various types of ILD, rs5743890_C exhibited a potential association with a reduced risk of ILD (*p* = 0.04, OR = 0.72, 95% CI = 0.53–0.99) and a significant association with a decreased risk of idiopathic pulmonary fibrosis (IPF) (*p* = 0.004, OR = 0.62, 95% CI = 0.44–0.86) (Fig. 2). Notably, the analysis revealed a substantial association between susceptibility to ILD and rs111521887_G (*p* < 0.00001, OR 1.48, 95% CI = 1.33–1.65) (Fig. 3), as well as the rs3750920_T allele (*p* < 0.00001, OR 1.34, 95% CI = 1.26–1.44) (Fig. 4). The details are shown in Table 3.

**Table 3:** Characteristics of individual studies on *TOLLIP* variants included in the meta-analysis of survival

References	Year	Country	Ethnicity	Disease	*n*	HR	95% CI	
**rs5743890**								
**Associations with transplant-free survival**								
Francesco Bonella^16^	2021	Germany	European	IPF	37	2.22	1.15	4.29
Chad A. Newton-1^20^	2019	America	European	IPF	499	1.41	1.10	1.81
Chad A. Newton-2^20^	2019	America	European	IPAF	233	0.65	0.37	1.13
Chad A. Newton-3^20^	2019	America	European	CTD-ILD	241	0.90	0.45	1.83
Brett Ley-1^17^	2017	America	European	CHP/IPF	142	0.94	0.42	2.10
Brett Ley-2^17^	2017	America	European	CHP/IPF	72	0.85	0.40	1.82
**Associations with survival**								
Chad A. Newton-1^20^	2019	America	European	IPF	499	1.46	1.08	1.98
Chad A. Newton-2^20^	2019	America	European	IPAF	233	0.64	0.34	1.23
Chad A. Newton-3^20^	2019	America	European	CTD-ILD	241	0.80	0.36	1.77
Brett Ley-1^17^	2017	America	European	CHP/IPF	142	0.91	0.39	2.14
Brett Ley-2^17^	2017	America	European	CHP/IPF	72	0.78	0.27	2.26
**rs3750920**								
**Associations with survival**								
Francesco Bonella^16^	2021	Germany	European	IPF	37	0.77	0.33	1.84
Takuma Isshiki^9^	2021	Japan	Asian	FILD	102	0.95	0.61	1.49
**rs111521887**								
**Associations with survival**								
Patrícia Caetano Mota-1^25^	2022	Portuguese	European	IPF	21	1.65	0.83	3.26
Patrícia Caetano Mota-2^25^	2022	Portuguese	European	IPF	2	5.99	0.74	48.20

CTD-ILD, connective tissue disease associated interstitial lung diseases; IPAF: Interstitial pneumonitis with autoimmune features; IPF: idiopathic pulmonary fibrosis; CHP: chronic hypersensitivity pneumonitis; FILD: fibrosing ILD

### Meta-analysis of the *TOLLIP* genotype and survival of patients with ILD

**Fig. 5:**
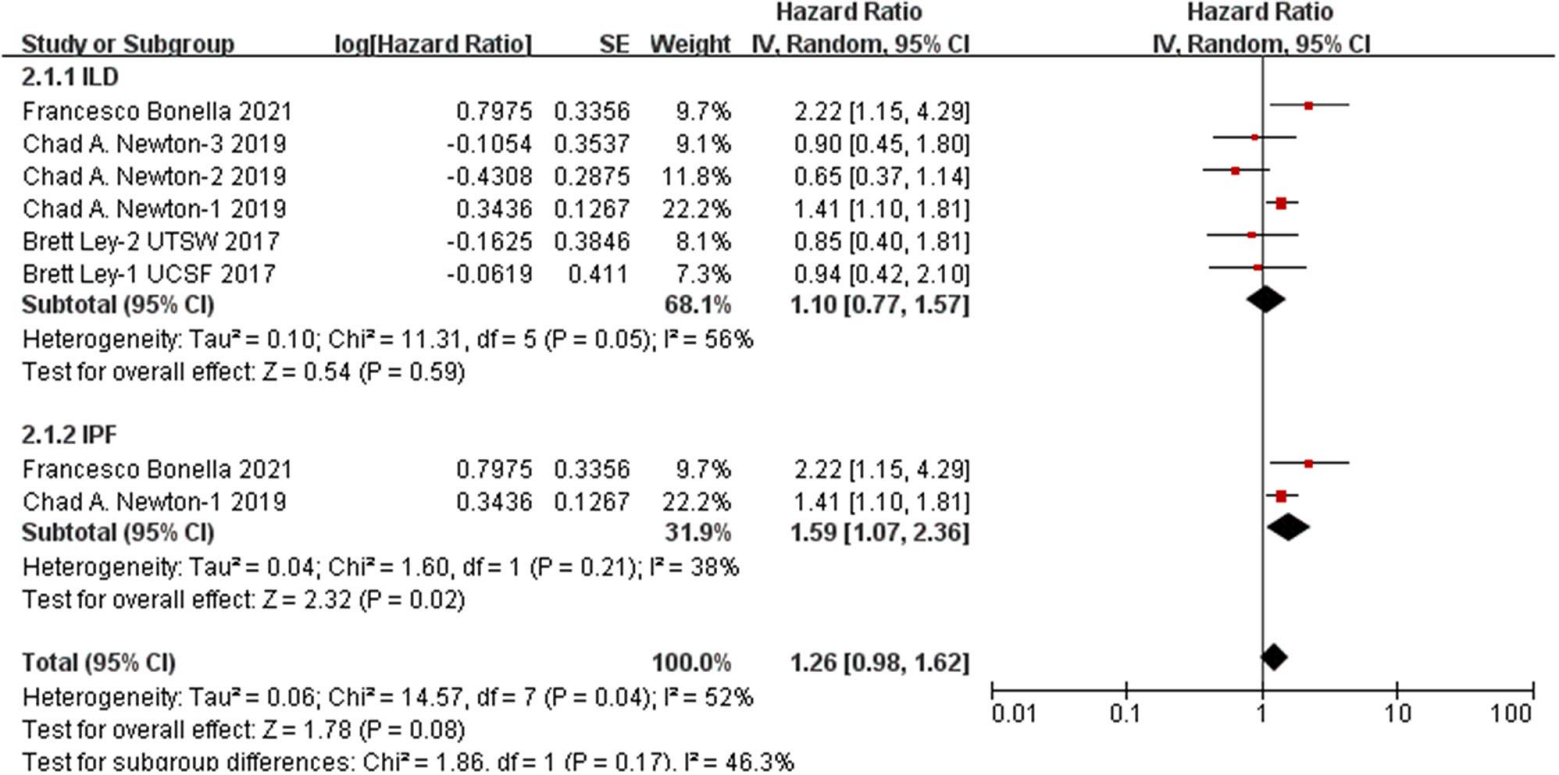
Analysis of the association between rs5743890_C and the survival of patients with ILD

**Fig. 6:**
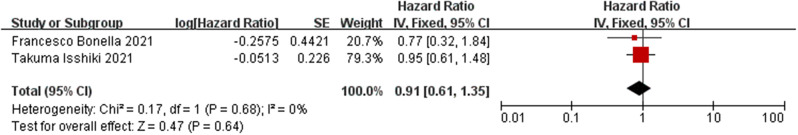
Analysis of the association between rs3750920_T and the survival of patients with ILD

**Fig. 7:**
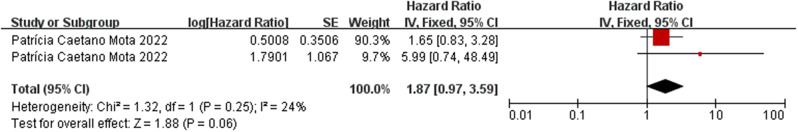
Analysis of the association between 111521887_G and the survival of patients with ILD

The presence of the *TOLLIP* rs5743890 SNP was not found to be associated with either overall survival or transplant-free survival among patients with ILD (survival: *p* = 0.59, HR: 1.10, 95% CI: 0.77–1.57; transplant-free survival: *p* = 0.27, HR: 1.14, 95% CI: 0.90–1.46). However, a stratified analysis revealed that rs5743890_C was significantly associated with worse survival in patients diagnosed with IPF (*p* = 0.02, hazard ratio (HR) 1.59, 95% CI = 1.07–2.36) (Fig. 5). No significant association was detected between rs3750920 and the survival of patients with ILD (*p* = 0.64, HR = 0.91, 95% CI = 0.61–1.35) (Fig. 6). Similarly, there was no significant association between rs111521887 and the survival of ILD patients (*p* = 0.06, HR 1.87, 95% CI = 0.97–3.59) (Fig. 7). The details are shown in shown in Table 4.

**Table 4:** Analysis of the association between the *TOLLIP* polymorphisms and survival of patients with ILD

Variants	Population	No. of studies	Test of association			Test of heterogeneity		
			HR	95%CI	*p* value	Model	*p* value	I^2^
**Associations with survival**								
rs5743890	Overall	6	1.10	0.77-1.57	0.59	RE	0.04	56%
	IPF	2	1.59	1.07-2.36	0.02	FE	0.21	38%
rs3750920	Overall	2	0.91	0.61-1.36	0.64	FE	0.67	0%
rs111521887	IPF	1	1.87	0.97-3.59	0.06	FE	0.25	24%
**Associations with transplant-free survival**								
rs5743890	Overall	5	1.14	0.90-1.46	0.27	FE	0.13	44%

HR, hazard ratio; CI, confidence interval; RE, R random effects model; FE, F fixed effects model

### Publication bias

Publication biases were evaluated with funnel plots. The funnel plots of any comparisons were symmetrical (Supplemental Figures S1-3). Therefore, publication bias was considered unlikely.

### Heterogeneity source and sensitivity analysis

Heterogeneity was observed, especially in the meta-analysis of rs5743890_C and susceptibility to ILD. Sensitivity analysis performed by excluding studies one by one did not reveal any apparent possible heterogeneity (data not shown).

## Discussion

To the best of our knowledge, this is the first inaugural meta-analysis conducted on the relationship between *TOLLIP* variants and interstitial lung disease (ILD). Our findings indicate that the frequency of rs5743890_C is reduced among ILD patients, whereas the frequencies of rs111521887_G and rs3750920_T are elevated. Specifically, our analysis revealed that rs5743890_C is associated with a decreased risk of ILD, while rs5743890_C is also linked to a decreased survival rate in individuals with IPF. On the other hand, rs111521887_G and rs3750920_T are associated with an increased incidence of ILD, although they are not significantly associated with ILD incidence or prognosis.

According to a prior genome-wide association study (GWAS) conducted in three stages, carriers of the minor allele of rs5743890 were found to have a reduced risk of developing IPF but a heightened risk of mortality [18]. Our meta-analysis examined the impact of rs5743890 on the prognosis of patients with ILD and included a study indicating that the minor allele of rs5743890 was associated with worse survival and disease progression in patients with IPF [16]. It is worth noting that, to incorporate all available data, the results included in our meta-analysis were not adjusted for other variables. We observed that, in another study included in our analysis, the association between the minor allele of rs5743890 and the survival of IPF patients was not statistically significant after we adjusted for variables such as age, sex, non-Hispanic white ethnicity, baseline forced vital capacity percent predicted, and baseline diffusion capacity of the lung for carbon monoxide percent predicted [20].

In this study, we identified the association between the minor alleles rs111521887 and rs3750920 and an increased incidence of ILD. However, these alleles were not found to be associated with the prognosis of ILD. Among the studies included in our analysis, only one small sample study did not reach the conclusion that the presence of rs111521887_G increased the incidence of ILD [14]. Moreover, the results of two genome-wide association studies (GWASs) supported that rs3750920_T was a risk factor for ILD [18, 19]. In a study involving a Japanese population, the frequency of rs3750920_T was lower in patients with fibrotic ILD, particularly among non-IPF patients [9]. Interestingly, although our meta-analysis revealed that rs3750920_T was not associated with the prognosis of ILD, previous studies have revealed a significant correlation between the response to N-acetylcysteine (NAC) therapy and rs3750920. These findings suggest that NAC may be an effective therapy for IPF patients with the rs3750920 TT genotype but may be associated with a trend toward harm in those with the CC genotype. Additionally, in a retrospective study involving patients with IPF and interstitial pneumonia with autoimmune features (IPAF), rs3750920 TT was more common in the positive antinuclear antibody (ANA) group, in which NAC exposure appeared to be beneficial for transplant-free survival [21].

Although these data could not be incorporated into the present study, it is worth noting that several other *TOLLIP* genetic variants have also been reported to be associated with the clinical phenotype of ILD. According to the previously mentioned three-stage genome-wide association study (GWAS), rs5743894_G significantly increased the risk of IPF. Furthermore, rs3829223_C, rs908225_T, rs5743944_A, and rs5743900_G also exhibited associations with susceptibility to IPF in both the primary GWAS and replication [18]. In a retrospective and prospective study, the presence of the rs5743899 GG genotype was associated with a rapid deterioration in forced vital capacity over time among patients diagnosed with chronic hypersensitivity pneumonitis (CHP) [22].

The precise roles of *TOLLIP* genetic variants in ILD remain enigmatic. *TOLLIP*, acting as an inhibitor, plays a pivotal role in downregulating the production of proinflammatory cytokines, promoting autophagy, and facilitating intracellular trafficking [23, 24]. A previous study revealed differences in *TOLLIP* expression among IPF patients bearing various *TOLLIP* minor alleles. Notably, *TOLLIP* expression was reduced in the lung tissue of minor allele carriers, decreasing by 20% in patients with rs5743890_C, 40% in those with rs111521887_G, and 50% in those with rs5743894_G [18]. The decreased *TOLLIP* expression observed in minor allele carriers suggested that *TOLLIP* deficiency may be involved in the pathogenesis of IPF.

This study is subject to several limitations. Significant heterogeneity was observed, particularly in the meta-analysis examining the relationship between rs5743890_C and susceptibility to interstitial lung disease (ILD). Although subgroup analyses were performed based on ILD classification, they did not entirely mitigate the observed heterogeneity, which could be attributed to the diverse study designs. It is important to recognize that multiple factors, such as age, sex, and smoking status, can influence the clinical phenotype of ILD. Consequently, the inherent heterogeneity of the study populations across the included studies might have contributed to the overall heterogeneity in our analysis. Additionally, certain subgroup analyses were limited by the limited number of included studies, and genotype distribution data were unavailable.

## Conclusion

This study represents the first meta-analysis investigating the relationship between *TOLLIP* genetic variants and clinical features of interstitial lung disease (ILD). The analysis revealed several key findings: the frequency of rs5743890_C was notably lower in ILD patients, while the frequencies of rs111521887_G and rs3750920_T were greater. Specifically, rs5743890_C was associated with a decreased risk of ILD incidence and emerged as a significant factor in the survival of patients with IPF. Conversely, the presence of 111521887_G and rs3750920_T was associated with an increased risk of ILD incidence, but no significant associations were identified regarding ILD prognosis. Further research is warranted to validate our findings and to comprehensively assess the impacts of *TOLLIP* genetic variants on ILD.

## Electronic supplementary material

Below is the link to the electronic supplementary material.

Supplementary Material 1

Supplementary Material 2

Supplementary Material 3

Supplementary Material 4

Supplementary Material 5

Supplementary Material 6

Supplementary Material 7

## Data Availability

The datasets supporting the conclusions of this article are included within the article (and its additional files).

## Abbreviations

Acronym: Full Form
TOLLIP: Toll-Interacting Protein
ILD: Interstitial Lung Disease
IPF: Idiopathic Pulmonary Fibrosis
CHP: Chronic Hypersensitivity Pneumonitis
CTD-ILD: Connective Tissue Disease-Associated ILD
SNP: Single Nucleotide Polymorphism
OR: Odds Ratio
CI: Confidence Interval
HR: Hazard Ratio
NOS: Newcastle–Ottawa Quality Assessment Scale
PROSPERO: International Prospective Register of Systematic Reviews
IPAF: Interstitial Pneumonitis with Autoimmune Features
