# Supplementary figures and images for: Associations between genetic variants of Toll-interacting proteins and interstitial lung diseases: a systematic review and meta-analysis
Source: Orphanet J Rare Dis. 2024 Nov 22;19:432. doi: 10.1186/s13023-024-03410-8 (PMC11583435; doi:10.1186/s13023-024-03410-8)

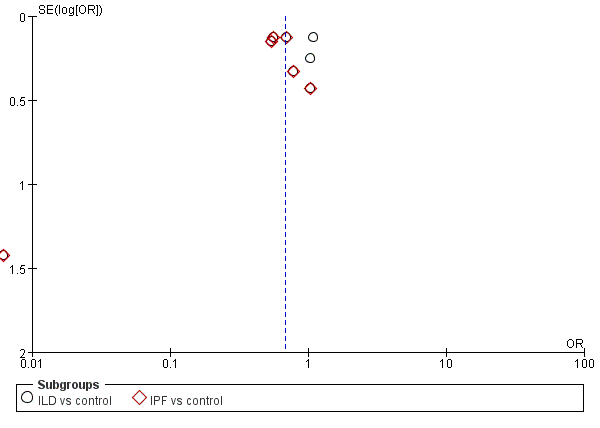

Supplement: Supplementary file 2 — Supplementary Material 2 [file 13023_2024_3410_MOESM2_ESM.png]

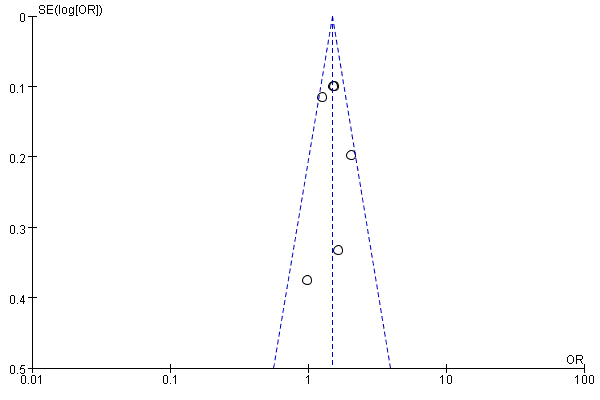

Supplement: Supplementary file 3 — Supplementary Material 3 [file 13023_2024_3410_MOESM3_ESM.png]

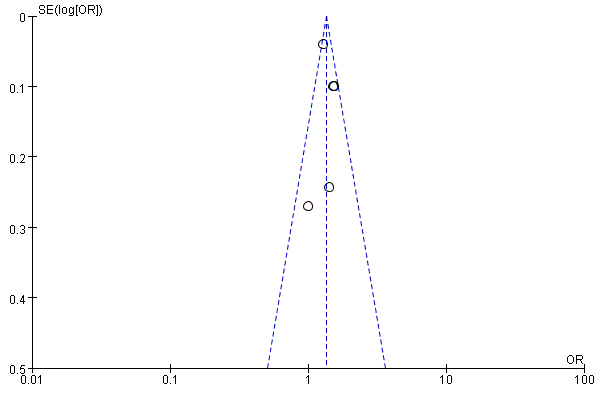

Supplement: Supplementary file 4 — Supplementary Material 4 [file 13023_2024_3410_MOESM4_ESM.png]
