# Supplementary material for: Associations between genetic variants of Toll-interacting proteins and interstitial lung diseases: a systematic review and meta-analysis
Source: Orphanet J Rare Dis. 2024 Nov 22;19:432. doi: 10.1186/s13023-024-03410-8 (PMC11583435; doi:10.1186/s13023-024-03410-8)
Supplement: Supplementary file 5 — Supplementary Material 5 [file 13023_2024_3410_MOESM5_ESM.pdf]

1 Lung Diseases, Interstitial
2 Pulmonary Fibrosis
3 interstitial and lung and disease
4 interstitial and fibrosis or pneumonitis or pneumonia or pneumopathy
5 diffuse and parenchymal
6 alveolitis
7 Bronchiolitis Obliterans or bronchiolitis and obliterans
8 good pasture and syndrome
9 granulomatosis
10 Histiocytosis or histiocytosis
11 Pneumoconiosis is or pneumoconiosis.
12 bagassosis
13 pulmonary and sarcoid
14 pulmonary and fibrosis
15 Wegener and granuloma
16 lung and purpura
17 bird or farmer or pigeon or avian or budgerigar and lung or disease
18 asbestosis or byssinosis or siderosis or silicosis or berylliosis or anthracosilicotic or silicotuberculosis
19 1 or 2 or 3 or 4 or 5 or 6 or 7 or 8 or 9 or 10 or 11 or 12 or 13 or 14 or 15 or 16 or 17 or 18
20 Scleroderma, Systemic
21 sclerodermas
22 Rheumatic Diseases
23 rheumatics
24 20 or 21 or 22 or 23
25 lung or pulmonary or respiratory
26 24 and 25
27 19 or 26
28 TOLLIP protein
29 tolls interacting protein
30 Toll-interacting protein
31 28 or 29 or 30
32 tollip
34 31 or 32
35 27 and 34
