# Supplementary material for: Associations between genetic variants of Toll-interacting proteins and interstitial lung diseases: a systematic review and meta-analysis
Source: Orphanet J Rare Dis. 2024 Nov 22;19:432. doi: 10.1186/s13023-024-03410-8 (PMC11583435; doi:10.1186/s13023-024-03410-8)
Supplement: Supplementary file 6 — Supplementary Material 6 [file 13023_2024_3410_MOESM6_ESM.pdf]

2023-12-19

Francesc Palau

Editor-in-Chief

Orphanet Journal of Rare Diseases.

Dear Editors:

We are submitting our manuscript titled "**Associations between Genetic Variants of Toll-Interacting Proteins and Interstitial Lung Diseases: A Systematic Review and Meta-Analysis**" for consideration in the *Orphanet Journal of Rare Diseases*. This original research, approved by all authors and not under consideration elsewhere, has no conflicts of interest.

Genetic variants of *TOLLIP* have been demonstrated to be associated with various chronic lung diseases. However, studies on interstitial lung disease (ILD) have produced conflicting results. Our study is the first meta-analysis exploring the relationship between *TOLLIP* genetic variants and interstitial lung disease (ILD) clinical features. We focused on variants rs5743890, rs111521887, and rs3750920. Our findings suggest that rs5743890_C is linked to a lower ILD incidence but poorer survival in IPF patients. Conversely, rs111521887_G and rs3750920_T are associated with higher ILD risk without impacting prognosis. These findings offer insights into the pathophysiology of ILD.

Acknowledging the scarcity of extensive research on ILD, we believe our study contributes valuable insights. Given your journal's history of publishing relevant research, we consider our manuscript a suitable addition.

We appreciate your consideration and look forward to your feedback. For any queries, please contact me.

Sincerely,

Lili Jiang, MD, PhD

Department of Pathology, West China Hospital,

Sichuan University, Guoxuexiang 37, Chengdu 610041,

Sichuan Province, China

Email: 879876047@qq.com
