# Supplementary material for: Associations between genetic variants of Toll-interacting proteins and interstitial lung diseases: a systematic review and meta-analysis
Source: Orphanet J Rare Dis. 2024 Nov 22;19:432. doi: 10.1186/s13023-024-03410-8 (PMC11583435; doi:10.1186/s13023-024-03410-8)
Supplement: Supplementary file 7 — Supplementary Material 7 [file 13023_2024_3410_MOESM7_ESM.docx]

Dear Editor:

We appreciate the opportunity to revise our manuscript " Associations between genetic variants of toll-interacting proteins and interstitial lung diseases: a systematic review and meta-analysis" (OJRD-D-23-01033). We thank the editor and reviewers for their constructive comments and suggestions. We have carefully considered all feedback and revised the manuscript accordingly, with changes marked in red. Our responses to the reviewers' comments are below:

**Reviewer #1:** Thank you for carrying out this excellent systematic review and meta-analysis, which allows us to be informed and obtain a more accurate estimate on the role of *TOLLIP* genetic variants in diffuse interstitial lung disease, based on the best available scientific evidence. Analyzes and synthesizes information in a practical way, based on the best available evidence. It uses a systematic and explicit method to select studies, and evaluates these articles critically and extracts those of most interest and analyzes them in order to obtain the most reliable information. This systematic review is rigorous in terms of the studies included, with quality criteria, focused on real problems, and tries to answer a clear question and presents the data in a way that helps to make decisions.

Response: We sincerely appreciate your positive feedback and the precise summary of our paper’s results. Thank you for your professional suggestions.
**Reviewer #2:** This is an interesting analysis of what has been published in the public domain regarding the role of *TOLLIP* mutations and the outcome of patients with Idiopathic Pulmonary Fibrosis (IPF). The problem with the manuscript is that it doesn’t place *TOLLIP* mutations within the bigger picture of what we know regarding the genetic basis of IPF. according to the literature it seems that overall, a genetic basis for IPF can be identified in about 25-30% of cases. The genetic picture is quite complex and *TOLLIP* mutations just a small part of what is an emerging picture. In order for the reader to understand this one has to have read or be part of a small group of people who follow this topic. The authors need to elaborate more why they think this particular mutation was examined and how it fits within the overall picture of what we know otherwise the meaningfulness of the exercise is lost. Why have you chosen to focus on susceptibility and prognosis which may less important than for example mutations affecting surfactant? in a picture where we still don’t know what is driving IPF and where the genetic underlying cause is not clear what is the importance of looking into this particular mutation? It doesn't seem all that important to be honest. Please elaborate more on these points and then why your findings are relevant in the overall picture of what we know about the etiology of the condition.

Response: Thank you very much for your insightful question. I agree that this aspect is crucial and was indeed underrepresented in the original manuscript. I appreciate the opportunity to address this gap and have now incorporated the relevant information into the revised manuscript.

Genetic factors have long been central to the study of interstitial lung disease (ILD). Genome-wide association studies and family-based research have identified several candidate genes involved in host defense, telomere maintenance, cell-cell adhesion, and signaling pathways. Among these, rare genetic variants, particularly those associated with alveolar stability and telomere biology, have been identified, although they account for only a small fraction of the risk for developing idiopathic interstitial pneumonia. But there has been no genetic variation associated with IPF results.

Significant attention has been given to the single nucleotide polymorphism (SNP) rs35705950 in the promoter region of the MUC5B gene, which has been linked to IPF susceptibility and survival, as demonstrated in previous meta-analyses. Furthermore, *TOLLIP* variants, first identified in a 2013 genome-wide association study by Imre Noth et al. in *Lancet Respiratory Medicine*, have been associated with IPF susceptibility and mortality, underscoring the importance of this gene in the disease's pathology.

*TOLLIP* and MUC5B are located in close proximity on chromosome 11p15.5 and play critical roles in immune response, bronchial, and alveolar fibrosis. While both are involved in host defense, *TOLLIP* is particularly relevant due to its association with inflammatory signaling. Subsequent research, including a 2015 study by Justin M. Oldham et al., has suggested a significant interaction between *TOLLIP* SNPs and NAC treatment, indicating that further exploration of *TOLLIP* could provide new avenues for therapeutic strategies in IPF.

However, current studies on *TOLLIP* SNPs have produced inconsistent results, likely due to geographic, racial, and sample size differences. Our meta-analysis was conducted to clarify these associations and explore the potential of *TOLLIP* as a therapeutic target in ILD. Our findings demonstrate a link between SNPs in *TOLLIP* and ILD susceptibility，but did not support a significant association between *TOLLIP* SNPs and ILD mortality, suggesting that more comprehensive data may be required to draw definitive conclusions.

I hope this explanation adequately addresses your concerns. I welcome any further feedback or suggestions for improvement, and I remain committed to refining my work. Thank you again for your valuable input.

Thanks for reviewer's· comments. and suggestions again. We have answered these questions in detail and revised our manuscript carefully. We would like to express our great appreciation again for giving us the opportunity to revise our manuscript. Thank you very much for your kind consideration.

Sincerely yours,

Lili Jiang，Department of Pathology, West China Hospital, Sichuan University, Chengdu, Sichuan, China
